# Visualizing Photodynamic Therapy in Transgenic Zebrafish Using Organic Nanoparticles with Aggregation-Induced Emission

**DOI:** 10.1007/s40820-018-0214-4

**Published:** 2018-07-04

**Authors:** Purnima Naresh Manghnani, Wenbo Wu, Shidang Xu, Fang Hu, Cathleen Teh, Bin Liu

**Affiliations:** 1 Department of Chemical and Biomolecular Engineering, National University of Singapore, 4 Engineering Drive 4, Singapore, 117585 Singapore; 2 Institute of Molecular and Cell Biology, Proteos Building, Biopolis Drive, Singapore, 138673 Singapore

**Keywords:** Nanomedicine, Photodynamic therapy, Transgenic zebrafish, Aggregation-induced emission, Organic nanoparticles

## Abstract

Photodynamic therapy (PDT) employs accumulation of photosensitizers (PSs) in malignant tumor tissue followed by the light-induced generation of cytotoxic reactive oxygen species to kill the tumor cells. The success of PDT depends on optimal PS dosage that is matched with the ideal power of light. This in turn depends on PS accumulation in target tissue and light administration time and period. As theranostic nanomedicine is driven by multifunctional therapeutics that aim to achieve targeted tissue delivery and image-guided therapy, fluorescent PS nanoparticle (NP) accumulation in target tissues can be ascertained through fluorescence imaging to optimize the light dose and administration parameters. In this regard, zebrafish larvae provide a unique transparent in vivo platform to monitor fluorescent PS bio-distribution and their therapeutic efficiency. Using fluorescent PS NPs with unique aggregation-induced emission characteristics, we demonstrate for the first time the real-time visualization of polymeric NP accumulation in tumor tissue and, more importantly, the best time to conduct PDT using transgenic zebrafish larvae with inducible liver hyperplasia as an example.

**Electronic supplementary material:**

The online version of this article (10.1007/s40820-018-0214-4) contains supplementary material, which is available to authorized users.

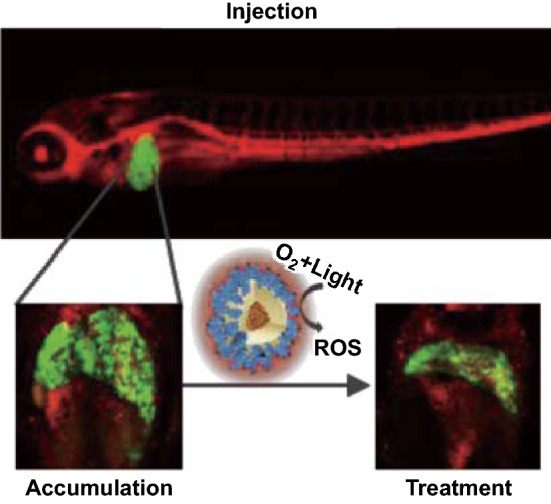

## Highlights

- The key novelty of this work is the creation of an in vivo model that can be used to effectively visualize image-guided photodynamic therapy. This allows fast screening of the performance of photosensitizers and their formulations.
- Transparent zebrafish larvae provide a visual understanding of bio-distribution of nanoparticles, thereby enabling smarter formulation strategies.

## Introduction

Photodynamic therapy (PDT) is a noninvasive triggered therapeutic modality, which involves the use of photosensitizer (PS) molecules capable of generating reactive oxygen species (ROS) upon light excitation for treatment of malignant and non-malignant tumors. Over the last three decades, several PSs have been developed and some of them have been successfully used to treat different kinds of cancers [1, 2]. PDT can also be combined with chemotherapy for synergistic therapeutic effect [3] and to achieve triggered drug release from vesicles [4]. Typically, upon light illumination, the formed PS triplet state releases energy to convert ambient oxygen molecules to reactive oxygen species (ROS) such as 1O_2_, H_2_O_2_, O_2_−^·^ and ^·^OH. The generated ROS can cause an increase in oxidative stress in cells. At elevated concentrations, ROS oxidize lipids, proteins and DNA, which leads to damage in various cell organelles and eventually cell death. Cancer cells operate at a sensitive oxidative threshold making them vulnerable to further increase in ROS [5]. Therefore, directly causing ROS to exceed the threshold concentration that is toxic to cells can kill cancer cells more easily as compared to that for normal cells [6]. Currently, PDT application potential is limited by low light penetration through tissue and poorly characterized tumor PS uptake. While the former can be addressed by chemiluminescence-induced ROS production [7, 8], so far there is no straightforward tool to characterize or visualize the PS uptake. However, the success of PDT is strongly dependent on PS versus light dosage, trigger time, PS distribution and oxygen concentration in the tumor microenvironment [9, 10]. Several clinical studies have characterized the PS dose and corresponding light dose for effective PDT in cancers of various organs [11, 12]. The oxygen concentration variation in the process of PDT has been modeled, simulated and validated in tumor spheroid models [13, 14]. Although the importance of quantifying PS accumulation has been realized [15, 16], a sensitive in vivo model to do the same has not been established. Real-time quantitative visualization of PS concentration in target tissue enables concentration-dependent trigger of the PS, thereby allowing unbiased evaluation of various PS molecules.

Theranostic cancer medicine aims at developing drug composites that can be successfully tracked in vivo post-delivery. A combination of therapeutic and imaging modalities for PDT enables real-time tracking, cancer characterization, targeted delivery, triggered drug release and pharmacokinetic profiling. Cancer imaging involves use of various modalities such as ultrasound imaging, computed tomography, magnetic resonance imaging, fluorescence imaging and nuclear imaging. Although various imaging modalities may be applied, high spatial resolution, low-cost and real-time display of fluorescence imaging provides a unique advantage [17, 18]. As most fluorescent drugs and dyes are small hydrophobic molecules, their selective accumulation in tumor tissue is contingent upon the circulation time. In order to impart stealth properties to drugs, their aggregates can be encapsulated into polymeric nanoparticles (NPs) with PEG polymer decorated on the surface, which provide a long circulation time leading to enhanced permeation and retention (EPR) in the tumor tissue [19]. Using fluorescent PSs encapsulated in polymeric NPs can enable real-time tracking of their fate, their selective tumor localization and consecutive PDT.

To continuously track the NP tissue concentration, we need strongly fluorescent, photostable and efficient PS NPs. Different from traditional PSs, which show quenched fluorescence and reduced photosensitizing capabilities in aggregate state, recently some PSs with aggregation-induced emission (AIE) characteristics have been developed to show bright fluorescence and strong capabilities in ROS production as NPs [20, 21]. AIE molecules generally possess rotor-like structures. They are almost non-emissive in molecularly dissolved state due to the free intramolecular motions which consume the excited-state energy. Upon aggregation, the restriction of molecular motion is able to activate the radiative decay channel to yield fluorescence. Although mice are considered as the gold standard for pharmacokinetic and pharmacodynamic studies, real-time quantitative tracking and direct visualization of NP delivery, their bio-distribution for optimized PDT in mouse models is cumbersome, inefficient and invasive. In order to study the NP organ distribution, the mice need to be killed and imaged since fluorescence imaging typically has a depth resolution of 3–5 mm [22]. In this regard, the transparent zebrafish larva model has shown to be effective for such studies [23, 24]. Zebrafish produces optically transparent embryos which are used by biologists to study development, genetics, environmental toxicology, pharmacology and cancer. Zebrafish genome is 70% homologous with the human genome, making it an attractive vertebrate model with high scalability [25]. In this study, we use the inducible transgenic zebrafish line which expresses the oncogene (*EGFP*:*kras*^V12^) under a liver-specific promoter that develops liver hyperplasia when subjected to drug mifepristone (RU-486) [26]. Continuous exposure to mifepristone at the adult stage can cause the progression of hyperplasia to a mix of hepatoblastoma, carcinoma, malignant ascites and metastasis. We, however, induce hyperplasia in transparent larval stage that enables visualization of PS NP distribution over time in the liver tissue followed by selective light treatment for initiating PDT. Owing to the presence of EGFP, the fluorescence from the hyperplastic liver can be used to monitor the change in liver tumor size. Upon introduction of the PS NPs into systemic circulation (retro-orbital injection), their progressive accumulation in liver tumors can be monitored, which facilitates treatment parameters optimization of PDT. In this paper, we demonstrate how the zebrafish liver tumor model enables optimized precise photodynamic therapy using AIE PS NPs as an example.

## Results and Discussion

**Scheme 1:**
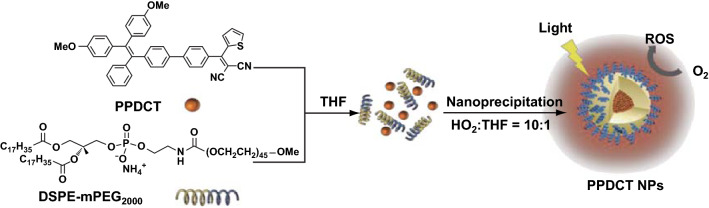
Nanoprecipitation of AIE photosensitizer PPDCT with DSPE-mPEG2000

The PS molecule of 2-((4′-(2,2-bis(4-methoxyphenyl)-1-phenylvinyl)-[1,1′-biphenyl]-4-yl)(thiophen-2-yl)methylene)malononitrile (PPDCT) [27] was selected because of its high fluorescence and good ROS production in the aggregate state. It exhibits twisted intramolecular charge transfer (TICT) and AIE properties (Fig. S1). Since the electron donor and acceptor groups of the PS molecule are linked by a single bond, when a polar solvent like water is introduced into the solvent, such molecules can undergo fast intramolecular electron transfer, which is accompanied by intramolecular donor–acceptor twisting around the single bond. However, as the fraction of water continues to increase, the AIE effect takes over to enhance the fluorescence. The hydrophobic PPDCT aggregates were encapsulated in amphiphilic polymer DSPE-mPEG_2000_ through nanoprecipitation [28]. PPDCT molecules and DSPE-mPEG_2000_ were dissolved in THF, which was mixed with water to give rise to a NP suspension at 160 µg mL^−1^ PPDCT concentration (Scheme 1). The molar absorption coefficient of the NPs was calculated to be 4.875 × 10^4^ L mol^−1^ cm^−1^, and relative fluorescence quantum yield of NPs is 10%, measured using 4-(dicyanomethylene)-2-methyl-6-(4-dimethylaminostyryl)-4*H*-pyran as the standard.

**Fig. 1:**
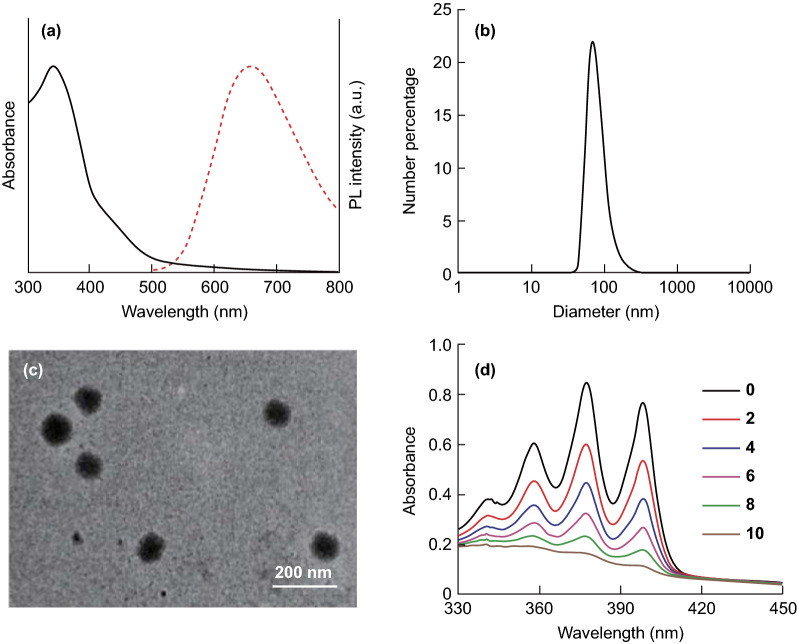
**a** UV–visible absorption (solid line) and photoluminescence (PL, dashed line) spectra of PPDCT-DSPE-mPEG NPs. **b** Number size distribution of PPDCT-DSPE-mPEG NPs. **c** TEM image of PPDCT-DSPE-mPEG NPs. **d** Absorbance decay of 64 µM ABDA in the presence of PPDCT NPs over 10 min of 0.15 W cm^−2^ white light

Liver is a NP filtration organ owing to its fenestrated vasculature. It possesses Kupffer cells and hepatocytes which are macrophages responsible for most of phagocytic activity in the liver. It has been shown that liver cells clear out big (> 60 nm) nanoparticles quicker than smaller ones [29]. Larger NPs may display increased uptake by liver due to their greater surface area for interaction with the cell membrane and surface receptors [30]. Hence, the NPs were designed to be 80 nm in size since liver is the targeted organ in the liver-tumor-bearing zebrafish (Fig. 1b, c). The PPDCT NPs show a broad absorption from 300 to 500 nm with an emission maximum at 660 nm (Fig. 1a).

The ROS production of the NPs was characterized by measuring the absorbance decay of indicator ABDA (9,10-anthracenediyl-bis(methylene)dimalonic acid) due to its reaction with ^1^O_2_ [31, 32] generated by the PS NPs in aqueous media under 0.15 W cm^−2^ white light (400–700 nm) excitation. As shown in Fig. 1d, under white light irradiation, the presence of PPDCT NPs at a fixed PS concentration can lead to gradual decrease in absorbance of ABDA (64 µM) in aqueous media, and the ^1^O_2_ generation of the AIE PS NPs is evaluated by the relative degradation of ABDA. Within 1 min, 15.8 nmol of ABDA could be degraded by 5 μM PPDCT NPs (based on molecules), which is significantly enhanced relative to Ce6 (5 μM) for which 12.2 nmol ABDA could be degraded under the same condition. This proves that PPDCT NPs could generate singlet oxygen in aqueous media with a relatively high efficiency.

**Fig. 2:**
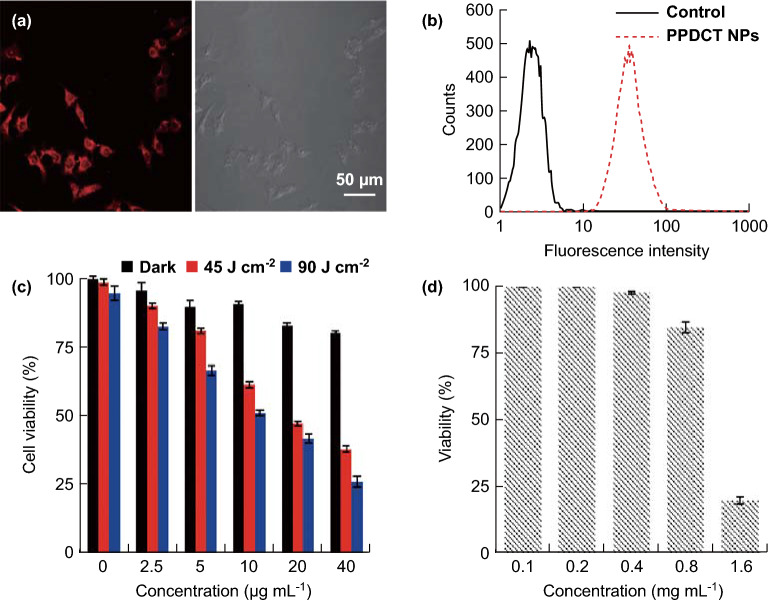
**a** Confocal image of HepG2 cells incubated with PPDCT NPs. **b** Cellular internalization of PPDCT NPs confirmed using flow cytometry. **c** MTT viability assay of HepG2 cells treated with PPDCT NPs and 0.15 W cm^−2^ white light (WL 0, 45 and 90 J cm^−2^). **d** Whole zebrafish embryo soaking viability for treatment with different PPDCT NP concentrations

The cell uptake of the PPDCT NPs was evaluated by incubating Hep G2 liver cancer cells with PPDCT NPs at 30 µg mL^−1^ PPDCT concentration for 24 h. The cells were imaged using confocal microscopy, and the fluorescence of the cells was subsequently confirmed using flow cytometry (Fig. 2a, b). The Hep G2 cells incubated with PPDCT NPs for 24 h were subjected to the MTT assay to determine the light-induced toxicity to the cells. As shown in Fig. 2c, 10 min of light exposure to cells incubated with 40 µg mL^−1^ NPs caused 70% of the cells to die. Once the therapeutic effect was established in vitro, we evaluated their safe working dose for zebrafish larvae.

**Fig. 3:**
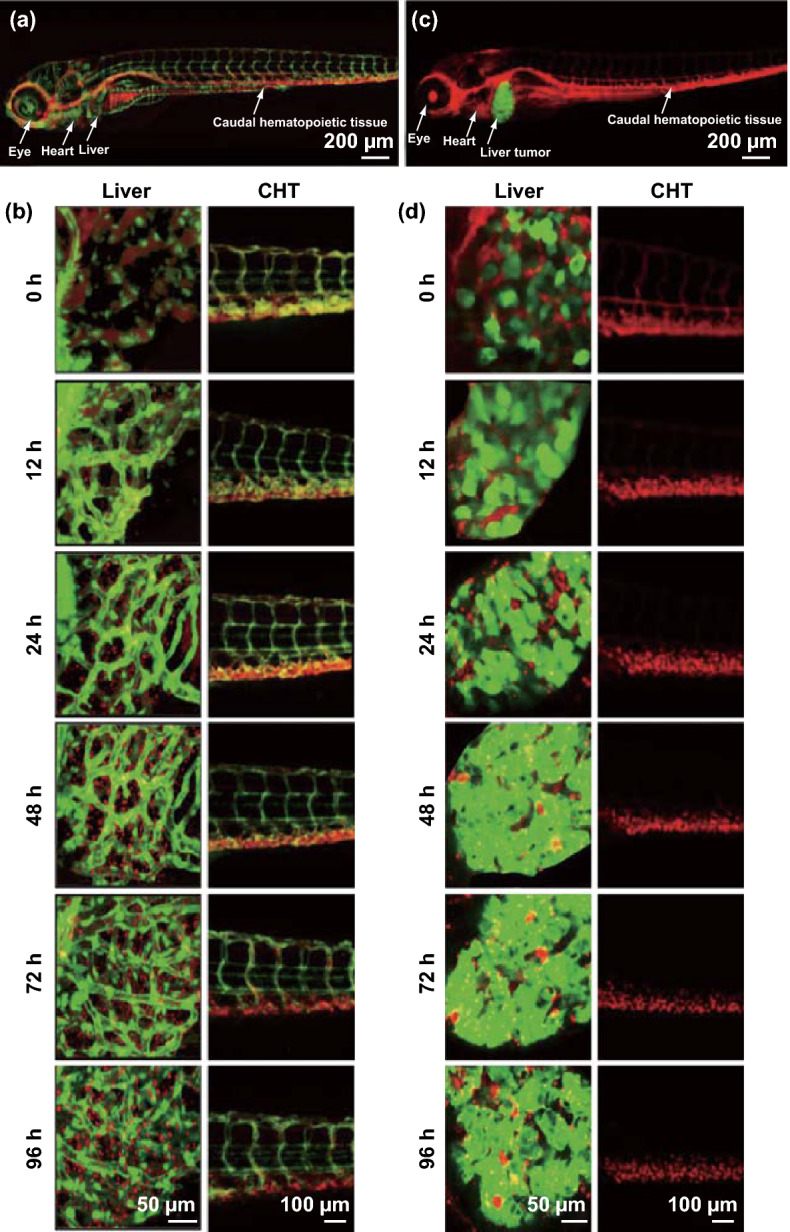
**a** Confocal image of fli:EGFP zebrafish larva injected intravenously with 0.8 mg mL^−1^ PPDCT NPs. **b** Uptake and breakdown of NPs over time span of 96 h in the liver and caudal hematopoietic tissue (CHT) of fli:EGFP zebrafish liver. **c** Confocal image of *EGFP*:*kras*^V12^ zebrafish larva injected intravenously with 0.8 mg mL^−1^ PPDCT NPs. **d** Uptake and breakdown of NPs over time span of 96 h in the liver and CHT of *EGFP*:*kras*^V12^ zebrafish liver. Confocal λ_ex_ = 488 nm, green fluorescent protein λ_em_ = 509 nm, PPDCT λ_em_ = 660 nm

Zebrafish larvae at 5 days post-fertilization (dpf) were soaked overnight in different concentrations of PPDCT NPs in a 96-well plate. Based on soaking, the working stock concentration for intravenous delivery was chosen to be 800 µg mL^−1^, at which about 90% larvae survived (Fig. 2d). This working concentration was chosen since intravenous delivery is a relatively benign treatment compared to whole embryo soaking. Approximately 5 nL of 800 µg mL^−1^ PPDCT NPs was injected intravenously into 7 dpf larvae through retro-orbital injection. In order to analyze PPDCT NP bio-distribution in zebrafish larvae, the NPs were first injected into the *fli1:EGFP* transgenic line of zebrafish. The *fli1:EGFP* zebrafish in Fig. 3a expresses the fluorescent protein EGFP in the endothelial cells of the blood vessel [33], enabling visualization of vessel development and PPDCT NP extravasation.

Confocal microscopy was used to image *fli1:EGFP* larvae (7 dpf), post-intravenous delivery. The PPDCT NPs accumulated passively in two regions—the caudal hematopoietic tissue (CHT) and the liver. The CHT is an equivalent for the bone marrow in zebrafish larvae and possesses most of the innate immune cells that can phagocytose PPDCT NPs. Progressive decrease in fluorescent labeling in CHT is used as an independent indicator of NPs biodegradation. The larvae were tracked up to 4 days post-injection. NPs in systemic circulation are depicted by yellow fluorescent signal where green fluorescent EGFP-labeled vessels co-localized with circulating red fluorescent PPDCT NPs. As shown in Fig. 3b, the red fluorescent NPs were initially in circulation within the vessels labeled with EGFP. As time progressed, the particles extravasated and penetrated into the developing liver. Exit from circulation is confirmed by distinct red fluorescent NPs that are away from neighboring GFP-positive vessels.

The liver blood vessel fenestrations and low blood flow rate [34] allowed blood carrying PPDCT NPs to interact with the hepatic cells. PPDCT NPs were recognized by hepatocytes as foreign materials and phagocytosed by scavenger receptors. Progressive NPs uptake in the liver was observed from 24 h post-injection (hpi). At 72 hpi, an absence in overlap of the EGFP and PPDCT NPs fluorescence indicated that almost all the NPs had extravasated. Progressive breakdown of internalized NPs followed from 96 h, which is suggested by the decrease in red fluorescence detected in CHT. A similar approach of tracking PPDCT NPs uptake was applied to the liver-tumor-bearing larvae (Fig. 3c). Seven dpf larvae were injected with the fluorescent PPDCT NPs and tracked over 4 days. In the hyperplastic liver tumor, successful uptake of red fluorescent PPDCT NPs is indicated by its detection in EGFP-positive liver cancer cells. Hence, confocal imaging using two different emission detection channels for EGFP emission at 509 nm and PPDCT emission at 660 nm identified co-localization of NPs in liver cancer cells, an unbiased in vivo assessment of uptake efficiency. Successful NPs internalization by liver cancer cells results in visualization of yellow fluorescence in the overlay image (Fig. 3d). The PPDCT red fluorescent intensity in the liver tumor was computed as a percentage of the EGFP intensity using ImageJ, to ascertain the concentration of the PPDCT NPs in the tumor tissue (Fig. S2). At 24 hpi, since most NPs were in circulation, the red fluorescence as a percentage of EGFP fluorescence in the liver was low. It gradually increases as the liver filters out the PPDCT NPs with time. Since the liver was hyper-proliferative and responsible for hepato-biliary excretion and degradation of the NPs, the uptake of PPDCT NPs from the single intravenous delivery would change significantly with time. At 96 hpi, the total concentration of PPDCT in continuously dividing liver cancer cells became suboptimal, such that there was less co-localization of red fluorescent PPDCT in EGFP-positive liver cancer cells (Fig. S2). Therefore, 48 hpi or 2 days post-injection (dpi) was chosen as the earliest trigger time after analyzing the uptake profile (Fig. 3d). We considered this period as the ideal time to conduct PDT testing in injected zebrafish to trigger an optimal therapeutic response because of the maximized cellular uptake of the PPDCT NPs.

**Fig. 4:**
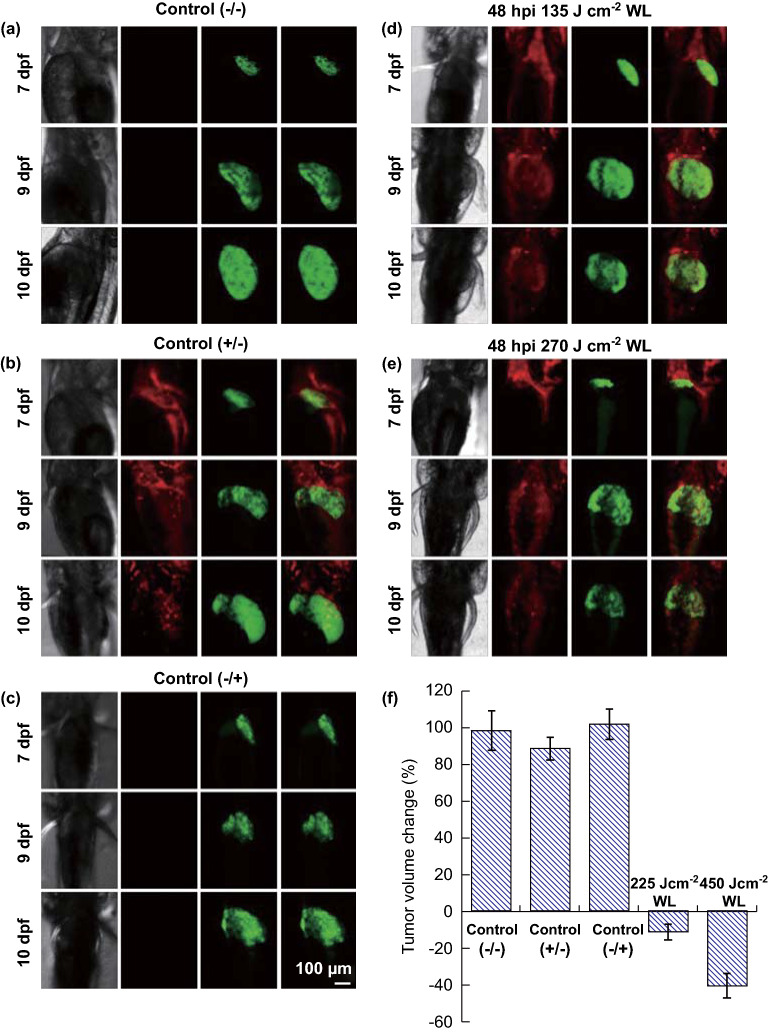
Change in liver tumor volume for control group: **a** without injection or illumination (−/−), **b** with injection and without illumination (−/+) and **c** without injection and with illumination (+/−). Change in liver tumor volume for group injected with NPs on 7 dpf and treated: **d** with 135 J cm^−2^ of illumination 9 dpf and **e** with 270 J cm^−2^ of illumination of 9 dpf. **f** Graph depicting the percentage of tumor volume change on 10 dpf relative to the volume on 9 dpf before light treatment for all five groups, n = 14; confocal *λ*_ex_ = 488 nm, green fluorescent protein *λ*_em_ = 509 nm, PPDCT *λ*_em_ = 660 nm

To assess their PDT potential in vivo, PPDCT NPs were intravenously injected into 7 dpf larvae and illumination was carried out 2 dpi. To trigger PDT, injected zebrafish were subjected to 135 and 270 J cm^−2^ white light treatments, respectively. Control groups for the experiment were untreated liver-tumor-bearing zebrafish, PPDCT NPs injected but non-illuminated group and liver-tumor-bearing zebrafish that underwent 270 J cm^−2^ white light illumination. Tumor-bearing volume in control groups was used as basis for determining therapeutic success (Fig. 4). The zebrafish confocal images were standardized by maintaining the microscope acquisition settings constant throughout the analysis, thereby capturing the change in GFP intensity. Specific delivery of white light to the liver was enabled by placing mounted zebrafish larvae behind an opaque sheet with a slit that exposed the zebrafish liver. The impact on liver hyperplasia was computed by measuring liver tumor volume from the threshold EGFP confocal images. The volume measurement process was automated using an image processing algorithm to prevent human bias and to achieve better accuracy. A peak detection method was employed on the histogram of these images to segment the tumor signal from the background [35, 36]. After obtaining a binary z-stack image which represents the tumor voxels, the volume of liver tumor was calculated from the voxels count. This algorithm was implemented in MATLAB code. An average 11 and 41% reduction in tumor volume was observed for 135 and 270 J cm^−2^ illumination, respectively, thereby confirming the importance of optimal light dose for therapy.

**Fig. 5:**
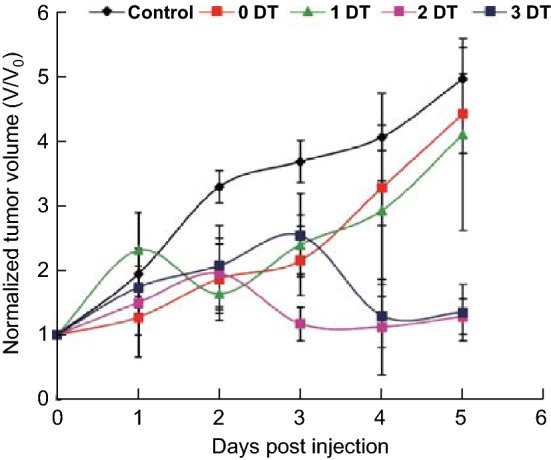
Changes in normalized tumor volume of larvae subjected to different days of light illumination; 0 dpi depicts 7 dpf larvae immediately after intravenous injection. 0-Day therapy (0 DT) refers to WL illumination on 0 dpi. *n* = 7

To demonstrate the role of accumulation in realizing effective PDT, the larvae were subjected to illumination at different days post-injection. The optimal illumination duration was chosen as 270 J cm^−2^ based on maximum tumor volume reduction achieved in Fig. 4. A strong correlation between PS accumulation in target tissue and effective therapy was observed as shown in Fig. 5. Normalized liver tumor volume was the ratio of liver tumor volume on any given day to the initial liver tumor volume. Hence, the 0-day tumor would give a normalized tumor ratio of 1. The control group with no injection or illumination showed a continuous increase in liver tumor volume. The injected larvae subjected to illumination immediately post-injection (0 DT) showed a negligible reduction followed by a continuous increase in tumor volume. The larvae treated 1 day post-injection (1 DT) showed minor therapeutic response as detected liver hyperplasia continued to grow aggressively on the following days. As PS accumulation in liver improved from day 2, therapeutic response was observed when illumination was carried out at 2 dpi and 3 dpi (*P* < 0.05). The therapeutic trend for larvae illuminated 2 dpi has been demonstrated through confocal images in Fig. S3. These results validate the need for optimized tumor drug load, accumulation-dependent trigger time and illumination duration for realizing the potential of a PS in PDT.

## Conclusions

In this study, we developed theranostic polymer-encapsulated NPs to carry out PDT. PPDCT was encapsulated in a polymeric shell, thereby imparting it with stealth properties. These NPs were shown to have effective PDT properties in vitro. Upon intravenous delivery, the PPDCT NPs passively accumulated in the hyperplastic liver of transgenic (*EGFP:kras*^*V12*^) zebrafish larvae. The PPDCT NP bio-distribution was profiled for normal (*fli:EGFP*) and liver-tumor-bearing larvae. Fluorescence-guided tissue accumulation data in zebrafish suggest the optimal time to conduct PDT. Effective duration of white light illumination was adjusted based on anticancer effect in treated zebrafish. This study demonstrates the importance of correlation between the tumor NP uptake, light dose and trigger time. There is a sweet spot for initializing triggered therapy which may vary based on the choice of nano-delivery system and the photosensitizer dose. Our research demonstrates how transparent zebrafish larvae can be used to study the effect of multiple light doses for PDT. Better in vivo understanding of therapeutic response will facilitate development of efficient triggered combination therapy like PDT-enhanced chemotherapy.

## Electronic supplementary material

Below is the link to the electronic supplementary material.

Supplementary material 1 (PDF 189 kb)
