# Supplementary material for: Visualizing Photodynamic Therapy in Transgenic Zebrafish Using Organic Nanoparticles with Aggregation-Induced Emission
Source: Nanomicro Lett. 2018 Jul 4;10(4):61. doi: 10.1007/s40820-018-0214-4 (PMC6199111; doi:10.1007/s40820-018-0214-4)
Supplement: Supplementary file 1 — Supplementary material 1 (PDF 189 kb) [file 40820_2018_214_MOESM1_ESM.pdf]

Supporting Information for

Visualizing Photodynamic Therapy in Transgenic Zebrafish Using Organic Nanoparticles with Aggregation-Induced Emission

Purnima Naresh Manghnani[1], Wenbo Wu[1], Shidang Xu[1], Fang Hu[1], Cathleen Teh[2], Bin Liu[1,] *

[1]Department of Chemical and Biomolecular Engineering, National University of Singapore, 4 Engineering Drive 4, 117585, Singapore

[2]Proteos Building, Institute of Molecular and Cell Biology, Biopolis Drive, 138673, Singapore

*****Corresponding author. E-mail: cheliub@nus.edu.sg (Bin Liu)

1 Experimental Section

1.1 Materials

9,10-Anthracenediyl-bis(methylene)dimalonic acid (ABDA), anhydrous dimethyl sulfoxide (DMSO), 3-(4,5-dimethylthiazol-2-yl)-2,5-diphenyltetrazolium bromide (MTT) and other chemicals were all purchased from Sigma-Aldrich and used as received without further purification. Tetrahydrofuran (THF) were dried by distillation using sodium or calcium hydride as drying agent. Photosensitizer PPDCT was synthesized according to reported literature [1]. Dulbecco’s Modified Essential Medium (DMEM) is a commercial product of Invitrogen. Milli-Q water was supplied by Milli-Q Plus System (Millipore Corporation, United States). Phosphate-buffer saline (PBS, 10×) buffer with pH = 7.4 is a commercial product of 1st BASE (Singapore). Milli-Q water (18.2 MΩ) was used to prepare the buffer solutions from the 10× PBS stock buffer. 1× PBS contains NaCl (137 mM), KCl (2.7 mM), Na_2HPO_4 (10 mM), and KH_2PO_4, fetal bovine serum (FBS) were purchased from Life Technologies.

1.2 Characterization

UV-Vis spectra were recorded on a Shimadzu UV-1700 spectrometer. Photoluminescence (PL) spectra were recorded on an Edinburgh FS5 spectrofluorometer. Average particle size and size distribution were measured by Zetasizer Nano ZS (Malvern Instruments Ltd, UK) at room temperature. The sample morphology was studied by transmission electron microscopy (TEM, JEM-2010F, JEOL, Japan).

1.3 Cell Culture

Human liver cancer cell-line Hep G2, was provided by American Type Culture Collection (ATCC). The cells were cultured in DMEM medium containing 100 μg mL^{-1} streptomycin, 10% heat-inactivated FBS, 100 U mL^{-1} penicillin, and maintained in a humidified incubator with 5% CO_2 at 37 °C.

1.4 Confocal Imaging

The cells were cultured in 6-well plates with glass slides and precultured overnight. Then the medium was replaced with fresh one and incubated with the PPDCT nanoparticles (30 µg mL^{-1}). The cells were fixed, mounted and imaged by confocal laser scanning microscope (CLSM, Zeiss LSM 800, Jena, Germany). For zebrafish imaging, the zebrafish larvae were immobilized by placing them in 1% low melting agarose in glass bottom dishes. Using an upright confocal microscope (CLSM, Zeiss LSM 800, Jena, Germany), the zebrafish larvae were imaged using 10X and 40X lens.

1.5 Flow Cytometry

In order to confirm cellular internalization, precultured cells in 6-well plates were incubated with PPDCT NPs (30 µg mL^{-1}) for 24 h. The cells were then trypsinized and fixed in suspension using 70% ethanol. The cells were then subjected to flow cytometric fluorescence measurement using 405 nm laser (Beckman Coulter CyAn ADP).

1.6 Cytotoxicity Studies

The metabolic activity of the cells was assessed by MTT assays. After incubation of the cells in DMEM medium overnight, the medium was removed, washed with PBS and the cells were incubated with PPDCT nanoparticles at different concentrations for 24 h and subjected to white light for different intervals. The cells were further washed with 1× PBS before the addition of 100 µL of MTT solution (0.5 mg mL^{-1}) into each well. After 3 h incubation, the MTT solution was removed and DMSO (100 µL) was added into each well. The absorbance of MTT at 570 nm was studied by microplate reader (Genios Tecan). The cells without any treatment were used as control.

1.7 Photodynamic Therapy in Zebrafish

For zebrafish photodynamic therapy, the zebrafish larvae were immobilized by placing them in 1% low melting agarose in glass bottom dishes. Specific delivery of white light of 0.15 W cm^{-2} to the liver was enabled by placing mounted zebrafish larvae behind an opaque sheet with a slit that exposed the zebrafish liver. Using an upright confocal microscope (CLSM, Zeiss LSM 800, Jena, Germany), the zebrafish larvae were imaged using 10× and 40× lens. Confocal λ_{ex} = 488 nm, Green fluorescent protein λ_{em} = 509 nm, PPDCT λ_{em} = 660 nm.

1.8 Zebrafish Line

Four to five pairs of zebrafish were placed in crossing tanks for spawning overnight. Embryos were settled to the bottom of the tank, and were collected using a sieve and transferred to petri dishes for embryo culture. They were screened, incubated at 27 °C , 0.4% CO_2 and grown in egg water (10% NaCl; 1.63% $MgSO_4{\cdot}7H_2O$; 0.4% $CaCl_2$; 0.3% KCl). At 22 h post fertilization, 1-phenyl 2-thiourea (PTU) was added to prevent melanin formation to yield optically transparent fish. *EGFP:kras*^{V12} transgenic zebrafish larvae were incubated with 5 µM of mifepristone in egg water with PTU. The embryos were imaged using confocal imaging

(Carl Zeiss LSM 800).

1.9 Microinjection

Local *in vivo* injection of the NPs intravenously was done using a nitrogen gas injector. The needle used for injection was made by pulling glass capillary tubes (O.D. 1.0 mm, I.D. 0.75 mm) in a needle puller which pulls the glass tubes into fine needles (20 µm). The NPs were filled into the needles which were loaded into the nitrogen gas injector. The injector was operated in continuous mode for the retro-orbital delivery of the NPs. The fishes were mounted in a mixture of 1% low melting agarose and 5% methyl cellulose for the injection and confocal imaging.

1.10 Statistical Analysis

Quantitative data were expressed as the mean ± standard deviation (SD). Statistical evaluations were made by comparing the area under the therapeutic trend curves in Fig. 5 by Student's t-test. P value < 0.05 was considered statistically significant.

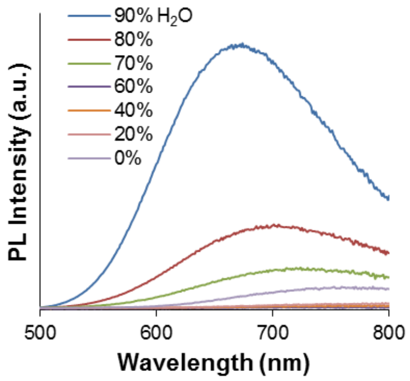

Fig. S1 PL spectral change of PPDCT molecules with varying water content in THF (vol%)

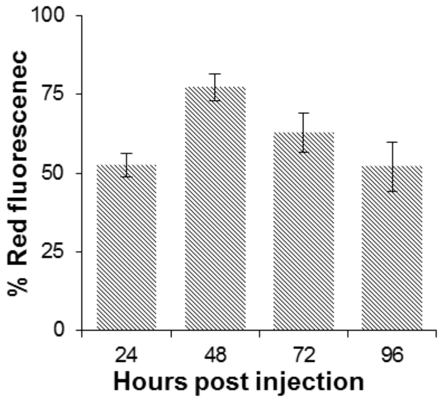

Fig. S2 The temporal change in PPDCT red fluorescence intensity in the liver tumor (computed as a percentage of the EGFP intensity), indicative of concentration change of the PPDCT NPs in the tumor tissue with time, n = 5

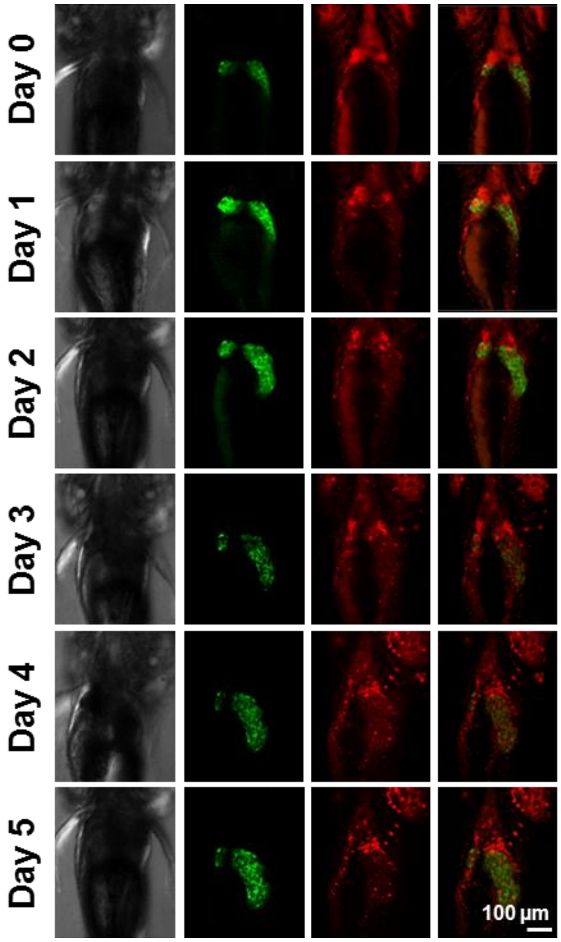

Fig. S3 Therapeutic trend for zebrafish liver tumor illuminated for 30 min 2 days post injection leading to decrease in EGFP signal on Day 3 followed by increase in tumor size due to continued oncogenic expression

Reference

[1] S. Xu, W. Wu, X. Cai, C.-J. Zhang, Y. Yuan, J. Liang, G. Feng, P. Manghnani, B. Liu, Highly efficient photosensitizers with aggregation-induced emission characteristics obtained through precise molecular design, Chem. Commun. **53**(62), 8727-8730 (2017). https://doi.org/10.1039/C7CC04864E
